# Characterization of *Alternaria alternata* alternariol monomethyl ether with a potential antiproliferative activity by topoisomerases inhibition; molecular docking and dynamic simulations

**DOI:** 10.1038/s41598-026-51757-8

**Published:** 2026-05-18

**Authors:** Ashraf S. A. El-Sayed, Moustafa O. Aboelez, Hend A. A. Ezelarab, Tassneim M. Ewedah, Ashraf F. El-Baz, Radwa Ashraf, Ayman Diab, Gehan Safwat, Amgad M. Rady, Rasha M. El-Mekkawy

**Affiliations:** 1 Enzymology and Fungal Biotechnology Lab, Botany and Microbiology Department, Faculty of Science, Zagazig University, Zagazig, 44519 Egypt; 2 Department of Pharmaceutical Chemistry, Faculty of Pharmacy, Sohag University, Sohag, 82524 Egypt; 3 Department of Medicinal Chemistry, Faculty of Pharmacy, Minia University, Minia City, 61519 Egypt; 4 Medicinal Chemistry Department, Faculty of Pharmacy, Minia National University, New Minia City, 61519 Egypt; 5 Pharmaceutics and Pharmaceutical Technology Department, Faculty of Pharmacy, Egyptian Russian University, Cairo, Egypt; 6 Department of Industrial Biotechnology, Genetic Engineering and Biotechnology Research Institute, University of Sadat City, 32897, Sadat City, Egypt; 7 Faculty of Biotechnology, October University for Modern Sciences and Arts, Giza, Egypt

**Keywords:** Alternariol monomethyl ether, *Alternaria alternate*, Anticancer activity, Topoisomerases inhibitor, Molecular docking, Biochemistry, Cancer, Chemical biology, Chemistry, Drug discovery

## Abstract

The drug resistance is one of the challenges in cancer chemotherapy, due to the development of different drug-efflux pumps that expels the drug out of the cells, thus, searching for new compounds with multiple targets in tumor cells, could be an affordable chemotherapy. Alternariol monomethyl ether (AME) was preliminary recognized as a cytotoxic compound, however, its availability and equivocal activity are the hurdles for further applications. *Alternaria alternata* EFBL-025, PV342518.1*,* endophyte of *Catharanthus roseus,* exhibited the highest AME productivity (550 μg/l). Chemically, the structure of putative compound of *A. alternata* was committed from the TLC, HPLC and LC-MS/MS, with molecular mass 274.2 m/z, and typical fragmentation pattern of authentic AME. The purified AME of *A. alternata* exhibited a substantial activity against the MCF-7 (IC_50_ 2.5 μM), HepG-2 (IC_50_ 3.5 μM), Caco2 (IC_50_ 3.9 μM), compared to OEC (IC_50_ 13.5 μM), i.e with selectivity indices 5.4, 3.9 and 3.5, respectively. The AME has a highest inhibitory activity of Topoisomerase II (IC_50_ 10.2 nM), than Topoisomerase I (IC_50_ 16.7 nM), with a noticeable ability to arrest the division of MCF-7 cells at the G2/M and S phases by 1.5 and 2 folds, respectively, compared to the control. The AME of *A. alternata* significantly induces the total apoptosis, early apoptosis and necrosis of MCF-7 by 20, 22 and 2.9 folds. The docking analysis showed that AME had a favorable binding affinity for topoisomerases I/II with binding scores -7.72 and -6.06 kcal/mol, normalized to camptothecin and etoposide that have binding scores -9.44 and -6.82 kcal/mol, respectively. The molecular dynamics simulations explored the reliable stability of protein-AME complexes.

**Supplementary Information:**

The online version contains supplementary material available at 10.1038/s41598-026-51757-8.

## Introduction

Chemotherapy is one of the most common approaches for combating the proliferation, invasion and metastasis of tumor cells, so, various compounds were used as a chemotherapeutic agent by targeting different metabolic pathways of tumor cells^1,2^. The miscellany of chemotherapeutic agents to selectivity target the different cellular machinery of tumor cells elaborates from the intricacy of their genetic machineries, signal transduction and metabolic pathway^3^. Recently, the insights of cell biology display a novel targets in apoptosis, angiogenesis, cell signal, and the growth factor modulating the tumor cells^4^. The overall activity of chemotherapeutic agents usually by interfering with synthesis and function of macromolecules like DNA, RNA, proteins, causing an ultimate cell death^5^. Based on their mechanisms, the chemotherapies were classified to alkylating agents, antimetabolites, and anti-micro tubular agents^2^. For the rapid division of tumor cells than normal cells, the tumor cells are more susceptible to the chemotherapeutic drugs. Currently, the most chemotherapies of solid cancers are the inhibitors of angiogenesis, histone deacetylase, mechanistic target of rapamycin, tyrosine kinase, topoisomerase I, II, tubulin polymerization and proteasome^1,5–7^.

Topoisomerase I and II inhibitors are one of the most efficient chemotherapies for various solid tumors^8,9^. Camptothecin derivatives are one of the well-known topoisomerase I inhibitors that was initially extracted from the bark of *Camptotheca acuminata*^10,11^, and different endophytic fungi inhabiting medicinal plants^12–15^. Topoisomerase I, responsible for easing the torsion of DNA, relaxing the DNA supercoiling by prompting the single-strand breaks during DNA replication, thus, inhibiting this enzyme suppress the DNA replication, transcription, with an ultimate growth arrest^16^. Etoposide is a semisynthetic derivative of podophyllotoxin glycoside, causing DNA damage by inhibiting DNA topoisomerase II, with a cellular cycle arrest at G2 phase^17–19^. The etoposide creates a ternary complex with DNA-topoisomerase II complex, blocking the activity of topoisomerase, promoting the cellular apoptosis, stopping the DNA relaxation, with a final cell death^20^, with a broad powerful activity against several aggressive malignancies^1^. However, the side effects of nausea, vomiting, low blood counts, low platelet, and scalp hair loss associated with the topoisomerase inhibitors chemotherapy^21,22^ are the main challenges. Etoposide resistance is a significant obstacle in treatment arising from multiple mechanisms that allow cancer cells to survive despite exposure to the drug, via alterations on chemistry of topoisomerase II, increased drug efflux and changes in DNA damage response^22,23^. Thus, searching for novel compounds with a potential higher affinity to inhibit the topoisomerases activity is one of the alternative promising approaches.

The genus *Alternaria* produces plethora of secondary metabolites with diverse biological activities as mycotoxins^24,25^. Among the *Alternaria* toxins, alternariol, alternariol monomethyl ether (AME), altenuene and tenuazonic acid belongs to the dibenzo-pyrone derivatives are reported as contamination in food products such as fruits, vegetables, cereals and grains^26^. Among the toxins of *Alternaria*, AME was reported as potential secondary metabolites inhibiting the topoisomerase activity^27^, however, the mechanism of cytotoxicity of this metabolite remains equivocal. The chemical structures of the AME, camptothecin, Topotecan and Etoposide were illustrated in Fig. S1. So, the objective of this study was to extract and chemically characterize AME from the metabolites of *Alternaria* spp, and exploring their mechanism of cytotoxicity, as confirmed from the molecular docking analysis and dynamic simulations.

## Materials and methods

### Plant samples collection, fungal isolation and identification

*Catharanthus roseus* samples were obtained from Botanical garden of Zagazig University at May/2024. The plant samples were collected, surface sterilized, sectioned, placed on plates of potato dextrose agar medium (200 g potato extract, 20 g glucose and 20 g agar/liter), for 10 days at 30°C^12^. The recovered fungi were purified and identified relied on microscopical features^28–30^. The potent fungal isolates were molecularly confirmed relied on their ITS sequence^31^. The genomic DNA was extracted by CTAP reagent^32^, used as a PCR template with the primers as described in our previous studies (El-Sayed et al., 2020, 2022, 2023). The PCR products were sequenced, non-redundantly BLAST searched, aligned by Clustal W^33^, and the phylogenetic relatedness was conducted by the neighborhood-joining method of 1000 bootstrap replications^34^.

### Screening for AME production by the endophytic fungal isolates

The productivity of AME by the recovered fungi was assessed on potato dextrose broth (PDB). One plug of 6 day old cultures of each fungus was inoculated to 50 ml PDB medium/250 ml Erlenmeyer conical flask, incubated for 10 days at 30 °C under static conditions. The cultures were filtered, extracted with ethyl acetate (1/1, v/v), dried by rotary evaporator at 45 °C, till oily residues^35^. Biological triplicates of each isolate were used. The residues were dissolved in 1 ml methanol, checked by TLC (pre-coated Silica gel 60 F254 plates), with methanol/water of 80:20 (v/v). The plates were illuminated at λ_254_ nm, the putative spots with the same color and mobility rate of authentic AME (Cat.#. HY-W013863), were considered. The silica gel spots of AME were carefully scraped-off, eluted, dissolved in 1 ml methanol (El-Sayed et al., 2024). The purity of eluted AME was assessed by HPLC (YOUNG In, Chromass) of RP-C18 column (Cat. #.959963-902), with mobile phase Methanol: distilled Water (80:20 v/v), with flow rate 1.0 ml/min, at wavelength λ_365_ nm. The identity of the AME was verified based on their retention time and peak area compared to the authentic AME.

### LC-MS/MS analyses

The molecular identity of the purified AME were analyzed by LC-MS/MS^36,37^, with Hypersil Gold aQ (C18 column), and the compounds were eluted by gradient of 2–98% of acetonitrile in 0.1 % formic acid, for 30 min at 0.2 ml/min. The ion trap was scanned from 300–2000 m/z in a positive-ion mode.

### Antiproliferative activity of the extracted AME

The antiproliferative activity of purified AME was evaluated towards the liver carcinoma (HepG-2) (ATCC, HB-8065), breast carcinoma (MCF7) (ATCC, HTB-22), human colorectal carcinoma cell line (Caco-2) (ATCC, HTB-37), in addition to the Oral Epithelial Cells (OEC) by the MTT reagent^38^. The cells were cultured on DMEM medium (Invitrogen, Life Technol.) of 10% FBS, and 10 μg/ml insulin. The plate was inoculated by 10^4^ cells/well in 100 µl medium, incubated for 12 h at 37 °C, with AME at different concentrations, and then incubated for 48 h. The MTT reagent was added, incubated for 6 h, and the developed complex was measured at λ_570_ nm, and the IC_50_ value was conveyed by AME concentration reducing the initial number of cells by 50%, compared to the control.

### Kinetics of DNA topoisomerase I, II inhibition for the AME

The activities of Topoisomerase I and II were determined according to the Kits manufacturer’s instructions (Cat.#.1015-1 and Cat.#. TG1001-1, TopoGen, Plasmid based, Florida, USA), based on their ability to convert the supercoiled DNA into relaxed DNA^39^. For Topoisomerase I, the reaction contains supercoiled DNA, 10x assay buffer and enzyme preparation in presence of different AME concentrations. The reaction was incubated for 30 min at 37 °C, checked by 1% agarose gel, and the DNA was visualized by UV-transilluminator. One unit of topoisomerase I was expressed by the enzyme concentration relaxing 1μmol supercoiled DNA in 1 min at 37ºC.

The topoisomerase II was determined by releases of minicircular DNAs by decatenation of the intertangled mass of kDNA. Topoisomerase II binds robustly to the kDNA networks, rapidly releasing the intact 2.5 kb monomeric rings that swiftly migrate to 1 % agarose gels, while, the extremely large mass of DNA fails to enter the gel. The reaction mixture contains 5 x reaction buffer, kDNA, topoisomerase II, different concentrations of AME in 20 μl total reaction, incubated for 30 min at 37 °C, stopped by 5x stopping buffer, and the relaxed/minicircular DNA was assessed by agarose gel. The IC_50_ value was represented by AME concentration suppressing the activity of topoisomerase II by 50%, compared to control.

### Apoptosis and cell cycle analyses of MCF-7 cells for extracted AME

The apoptosis process of MCF-7 cells for AME was detected by Annexin V-FITC Detection Kit (Cat.#.K101-25). The cells were seeded into a 96-well plate culture (2x10^6^ cells/well), incubated for 12 h at 37 °C, amended with AME doses, and incubated for 48 h under standard conditions. The cells were harvested, washed with PBS, supplemented with 1X Annexin buffer, Annexin V-FITC, PI, incubated for 15 min at room temperature according to the manufacturer’s instructions. Annexin V-FITC binding was measured at Ex, 488 nm and Em, 530 nm.

The cell cycle of MCF-7 cells was assessed by the propidium iodide (PI) Flow Cytometry (Cat#. ab139418). Briefly, the cells were incubated for 12 h at 37 °C, amended with the tested AME at its IC_25_ values, re-incubated for 48 h, the cells were harvested, and fixed by 70 % ethanol for 2 h at 4°C. The cells were rehydrated with PBS, stained with PI solution for 30 min in the dark, and the DNA contents were measured by flow cytometry (Ex λ_493_ nm, and Em λ_636_ nm). The cell cycles phases (G0/G1, S and G2/M) were evaluated by FACS Software.

### Molecular docking and dynamic simulation analysis

The molecular affinity of putative *A. alternata* AME to bind with Topoisomerase I and II was assessed from the docking analysis. The sequences of human Topoisomerase I and II were derived from the protein database with PDB codes as **1T8I** and **3QX3**, respectively^40,41^. The *in silico* analysis were conducted *via* re-docking of the co-crystallized compound as co-localized ligands (camptothecin and etoposide) within the active binding regions of topoisomerases I and II, respectively. Camptothecin and etoposide re-fitted drugs and naturally coexisted ligands’ root means square deviation (RMSD) values were 1.47 and 1.08 Å, respectively, ensuring the viability of the docking protocol. Topotecan as a common topoisomerase I inhibitor as a positive reference, was docked within topoisomerases I and II active sites.

The molecular dynamic simulations of the AME with topoisomerase I and II was conducted, by iMODs server^42–44^. The different parameters such as variance, eigenvalues, B-factor, elastic network, and covariance maps were extracted. The input docked PDB files were uploaded to iMODs, the initial limits were left at their recorded standard values when the outcomes were displayed. The normal mode analysis (NMA) was utilized alongside the docked complex’s coordinates, to investigate both molecular mobility and structural pliability.

### Statistical analysis

Biological triplicates of each experiment were conducted. The results were represented by the means ± SD.

## Results

### Isolation, identification of AME-producing endophytic fungal isolates of *C. roseus*

**Fig. 1:**
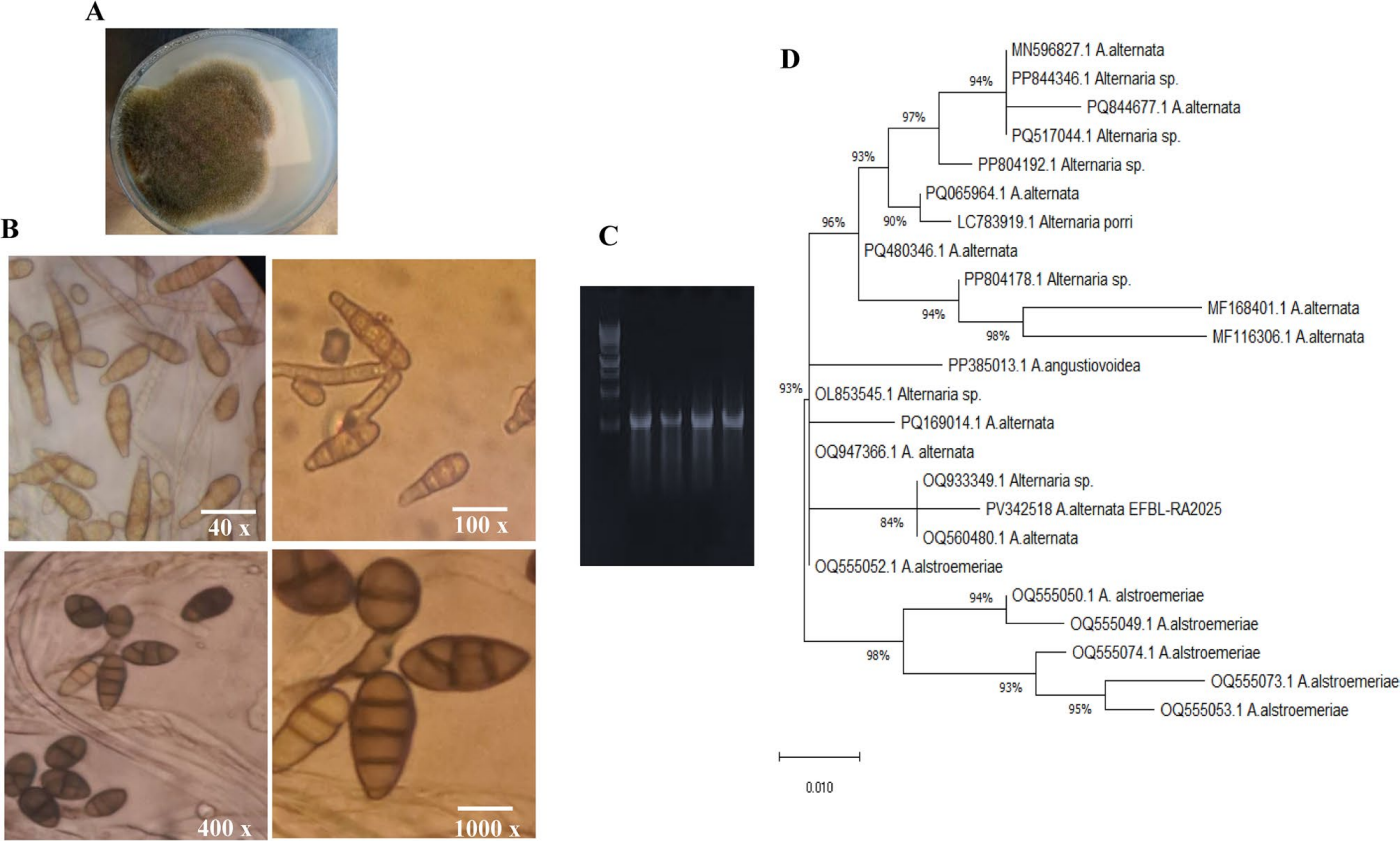
Morphological and molecular identification of *Alternaria alternata,* an endophyte of *Catharanthus roseus,* as the most potent AME producing isolate*.* (**A**), Plate culture on PDA after 7 days. (**B**), Conidial ontogeny of *A. alternata* at 40, 100, 500 and 1000 X. (**C**), PCR amplicons of the ITS region of *A. alternata*. (**D**), The phylogenetic tree of the ITS sequences of *A. alternata* by the Maximum Likelihood method with MEGA X software package.

Twenty fungal isolates were recovered from the leaves of *C. roseus* on PDA medium, and morphologically recognized to the species level^37^. These fungal isolates were grown on DDB, incubated for 10 days, at 30 °C, and their metabolites were recovered, and checked by HPLC. Among the recovered fungi, *Alternaria alternata* was the most potent AME producing isolate (400 μg/l), as quantified by TLC and HPLC, normalized to authentic AME. Based on the microscopical features (Fig. 1), the tested fungal colony appears grey-black with velvety appearance, branched conidiophores with septated hyphae, with light-dark green ellipsoidal conidia, with/without longitudinal septa. The morphological features of the current isolate closely follow the features of *A. alternata* as described^30^. The identity of the fungal isolate was molecularly confirmed by the ITS sequence, with genomic DNA as a PCR template. The PCR amplicon of the ITS region was 600 bp (Fig. 1), sequenced, and non-redundantly BLAST searched on NCBI database, gave 99.8 % similarity with the ITS sequences of *A. alternata,* of 100% query coverage and zero E-value. The ITS sequence of the isolate “*A. alternata* EFBL-RA025 was deposited to the Genbank with accession # PV342518.1. From the phylogenetic analysis of the ITS sequences, two clusters (I, II) were appeared. The isolate *A. alternata* EFBL-RA2025 gave 99.8 % similarity with *A. alternata* of accession # OQ933349.1, OQ560480, OQ947366, PQ169014, MF116306, MF168401, PQ480346, PQ065964, PQ844677 and PQ844677, with zero E-value and 100% coverage. Consequently, from the morphological and molecular fingerprint, the current isolate was identified as *A. alternata.*

### Chromatographic and LC-MS/MS analysis of the extracted AME

**Fig. 2:**
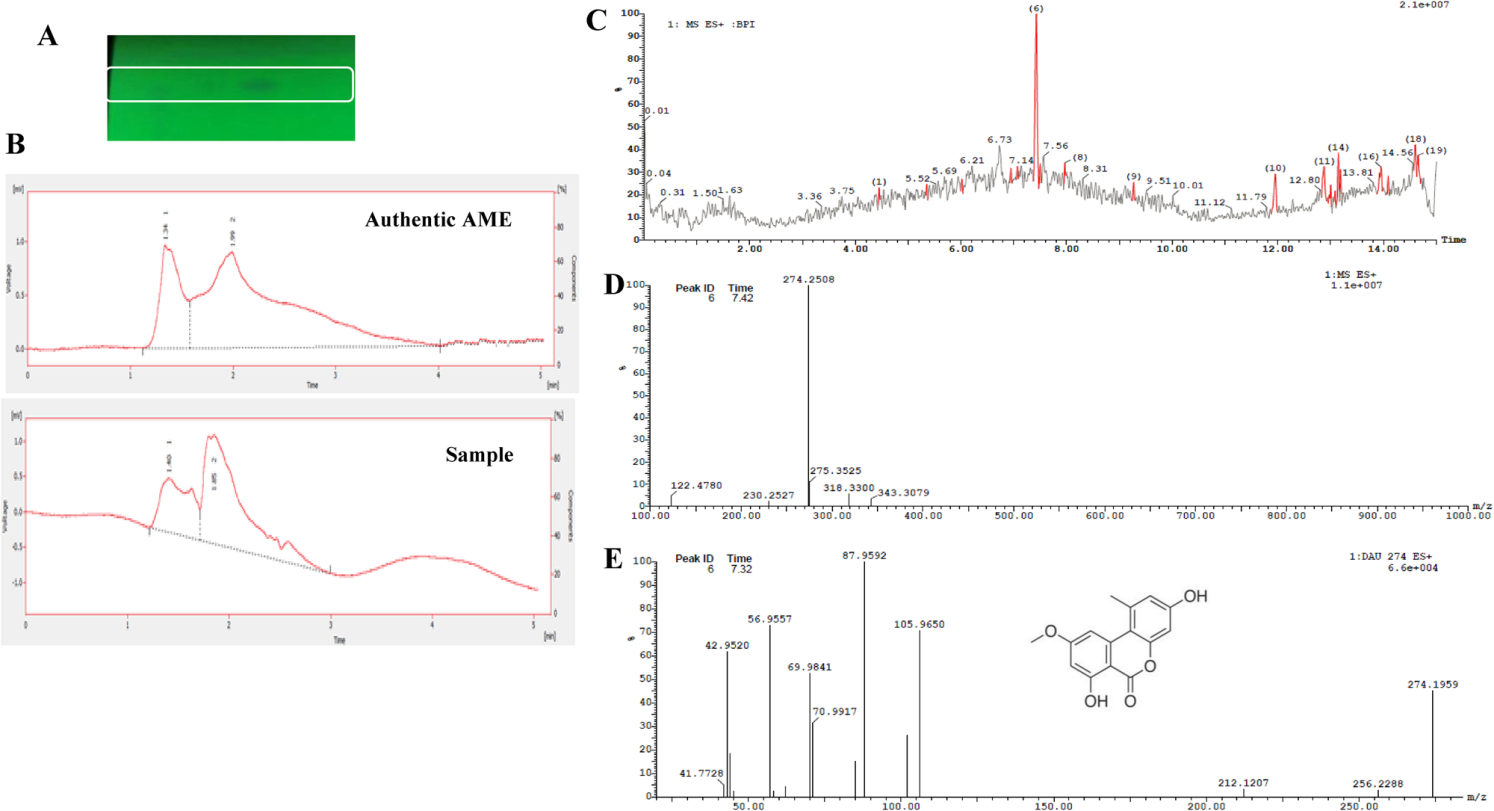
Chromatographic and spectroscopic analysis of purified AME from *A. alternata*. The fungal cultures were extracted with ethyl acetate, the extracts were dried, fractionated by TLC (**A**), and HPLC (**B**). The chemical identity of the putative AME was determined by LC-MS/MS. C, The Total Ion chromatogram of the TLC-purified putative compound. D, The molecular mass of the parent molecule of AME by LC/MS (274.2 m/z). E, The molecular fragmentation of the parent molecule by LC-MS/MS (256.2, 105.9, 87.9, 70.9, 69.9, 56.9, 42.9, and 41.7 m/z).

The chemical identity of the presumed spot of *A. alternata* AME on TLC was verified by HPLC and LC-MS/MS analyses. The silica gel spots of the putative AME, with the identical color and mobility rate of the authentic one was extracted, eluted, and their purity was checked by HPLC. From the HPLC profile (Fig. 2), the eluted compound has the same retention time of the authentic one, at 1.89 min, under the same conditions, ensuring the chemical proximity of the sample as AME. From the HPLC, the concentration of purified AME of *A. alternata* was 400 μg/l as calculated from the peak area of known concentration of the authentic AME.

The structure of the eluted compound was verified by LC-MS/MS analysis. From the total ion chromatogram, the major fraction was reported at 7.5 min, with molecular mass to charge ratio of parent molecule 274.2 m/z that was typical to the authentic AME. Furthermore, from the MS/MS analysis, the parent molecule of AME was fragmented by collision energy of 35 eV, and the molecular mass of the fragments were 256.2, 105.9, 87.9, 70.0, 69.9, 56.9, 42.9 and 41.7 m/z, that distinctive to the pattern of authentic AME fragmentation. From the LC-MS/MS analysis, the putative compound has retention time and molecular ion peak at 7.5 min and 274.2 m/z, with the same fragmentation pattern to the authentic one, corresponding to the structure C_15_H_12_O_5_. Thus, from the HPLC, LC-MS/MS analysis, the putative sample has the same polarity, molecular mass and fragmentation pattern of the authentic AME, ensuring their chemical identity as AME.

Prior to the cytotoxic analyses, the purity of the TLC-purified compound was checked again by the HPLC, as the area of the main peak/sum of area of main peak and other peaks x100. The yield of purified AME for the subsequent cytotoxic analysis was 480 μg/l, with total purity 81 %.

### Antiproliferative, and dynamics of Topoisomerase I and II inhibition by *A. alternata* AME

**Fig. 3:**
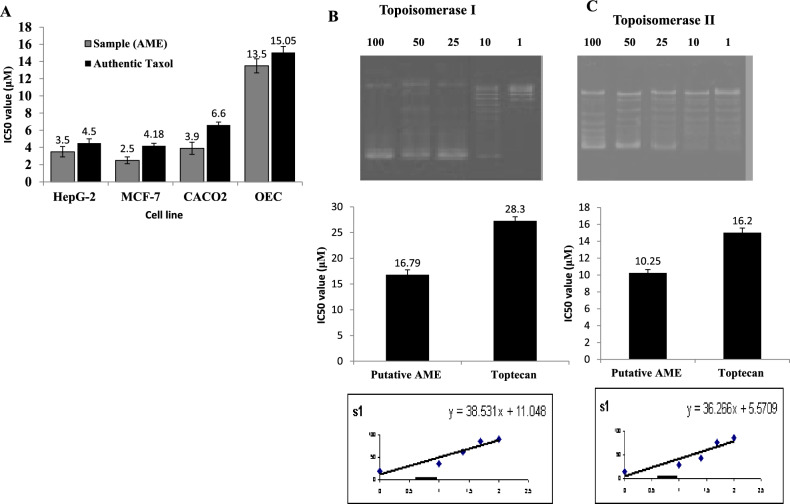
Antiproliferative, and anti-Topoisomerase I and II activities of the purified AME of *A. alternata.* A*,* The anti-proliferative activity of the purified AME towards the different cell lines (MCF7, HepG-2 cells,Caco2, and normal OEC cells), compared to Taxol as a positive control. Kinetics of Topoisomerase I (**B**) and Topoisomerase II (**C**), inhibition at different concentrations of AME by *A. alternata* as shown from agarose gel (upper panel), and IC50 value (lower panel).

The activity of the purified *A. alternata* AME against the different cell lines such as HepG-2, MCF-7, and Caco2 was evaluated compared to the OEC cells. The purified compound was added to the growth medium of the tumor cells at different concentration, then the cells viability were assessed. Practically, the viability of tumor cells was strongly reduced in a concentration-dependent manner by the *A. alternata* AME*.* From the IC_50_ values (Fig. 3), the purified AME of *A. alternata* had the highest activity against the MCF-7 cell (2.5 μM), HepG-2 (3.5 μM) and Caco2 (3.9 μM) compared to the OEC cells (13.5 μM). Overall, the purified AME of *A. alternata* had a higher activity towards the tested cell lines, compared to Taxol as standard anticancer drug by 1.2 folds, for the tested cells. The effective concentrations of the purified AME and authentic Taxol for OEC, as normal cells were 13.5 and 15.05 μM, respectively. So, the selectivity index of the purified AME of *A. alternata* was about 3.9 fold for the tested tumor cell lines, compared to the OEC as normal cells.

The inhibitory effect of the purified AME of *A. alternata* for the topoisomerase I and II was assessed. The AME were added to the standard reaction of Topoisomerase I and II with the supercoiled circular DNA, and the relaxed DNA was estimated. From the dynamics of the enzymes inhibition (Fig. 3), the IC_50_ values of the purified *A. alternata* AME towards topoisomerase I and II were 16.7 nM and 10.2 nM, respectively. While, the IC_50_ value of Topotecan for topoisomerase I and II was 27.2 and 15.02 nM. So, the affinity of *A. alternata* AME to inhibit topoisomerase II was about 1.6 folds higher than the effect on topoisomerase I.

### Cell cycle and apoptosis analyses of MCF-7 cells in response to the AME

**Fig. 4:**
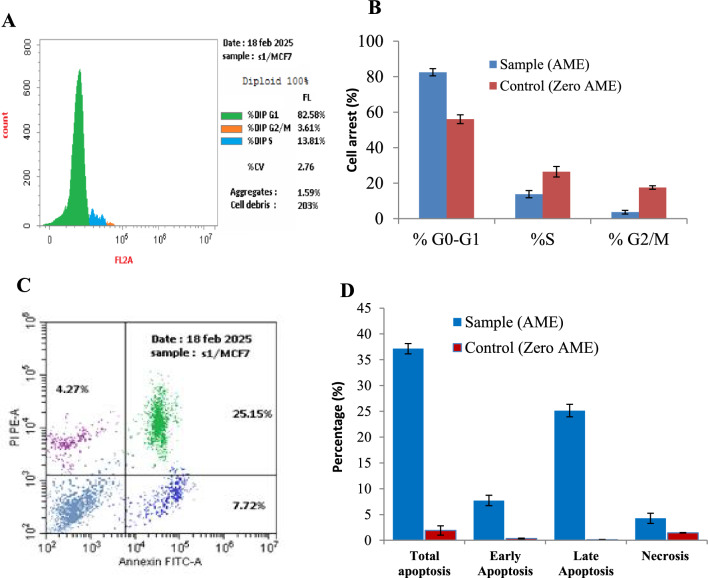
Cell cycle and apoptosis analyses of the MCF-7 cells in response to AME of *A. alternata*. The cells were treated with the extracted AME at their IC25, the cell cycle and apoptosis were measured after 48 h of incubation. The cell cycle of MCF-7 cells treated with AME (**A**), and overall growth arrest (**B**). The apoptosis of the control MCF-7 cells treated with AME (**C**), and the overall apoptosis percentage (**D**).

The cell cycle of MCF-7 cells in response to AME of *A. alternata* was analyzed by adding the compound at its IC_25_ values (1.1 μM), incubated, and the % of G0-G1, S and G2-M cells were assessed. From the results (Fig. 4), the highest growth arrest was observed at the G2/M phase by about 96.4% reduction in growth compared to 82.5 % reduction of control cells (without drug). The cellular DNA contents of the MCF-7 in response to the AME of *A. alternata* was 3.62 %, compared to control cells (17.53%), i.e 4.9 folds reduction of MCF-7 growth upon addition of AME, compared to the control. As well as, the putative compound had an obvious inhibitory effect on the cell cycle of MCF-7 at their S-phase, by about 2 folds (13.8%), compared to the control cells (26.44%), while, there is no an obvious effect to the compound on the G0-G1 phases of the tumor cells. So, from the cell cycle analysis, the AME of *A. alternata* had a significant inhibitory effect to the cell cycle at their G2/M phase and S-phase, suggesting the interference with the machinery of mitotic division and DNA synthesis of the cells.

The potency of the purified putative AME of *A. alternata* to induce the apoptosis process of the MCF-7 cells was assessed by Annexin V-PI assay. From the flow cytometry results (Fig. 4), the AME of *A. alternata* had a significant effect on inducing the apoptotic processes of the cells, compared to control cells. The putative compound induces the total apoptosis, late apoptosis, and early apoptosis of MCF-7 cells by about 37.14, 25.15 and 7.72 %, compared to 1.91, 0.11 and 0.24% of the control, respectively. So, upon addition of AME, the total apoptosis cells, early apoptosis and necrosis of the MCF-7 were increased by about 20, 22 and 2.9 folds, compared to control cells. Excitingly, upon addition of AME the late apoptosis of the MCF-7 cells was increased by about 228.6 folds, compared to the control cells.

### Molecular docking and dynamic simulation

**Fig. 5:**
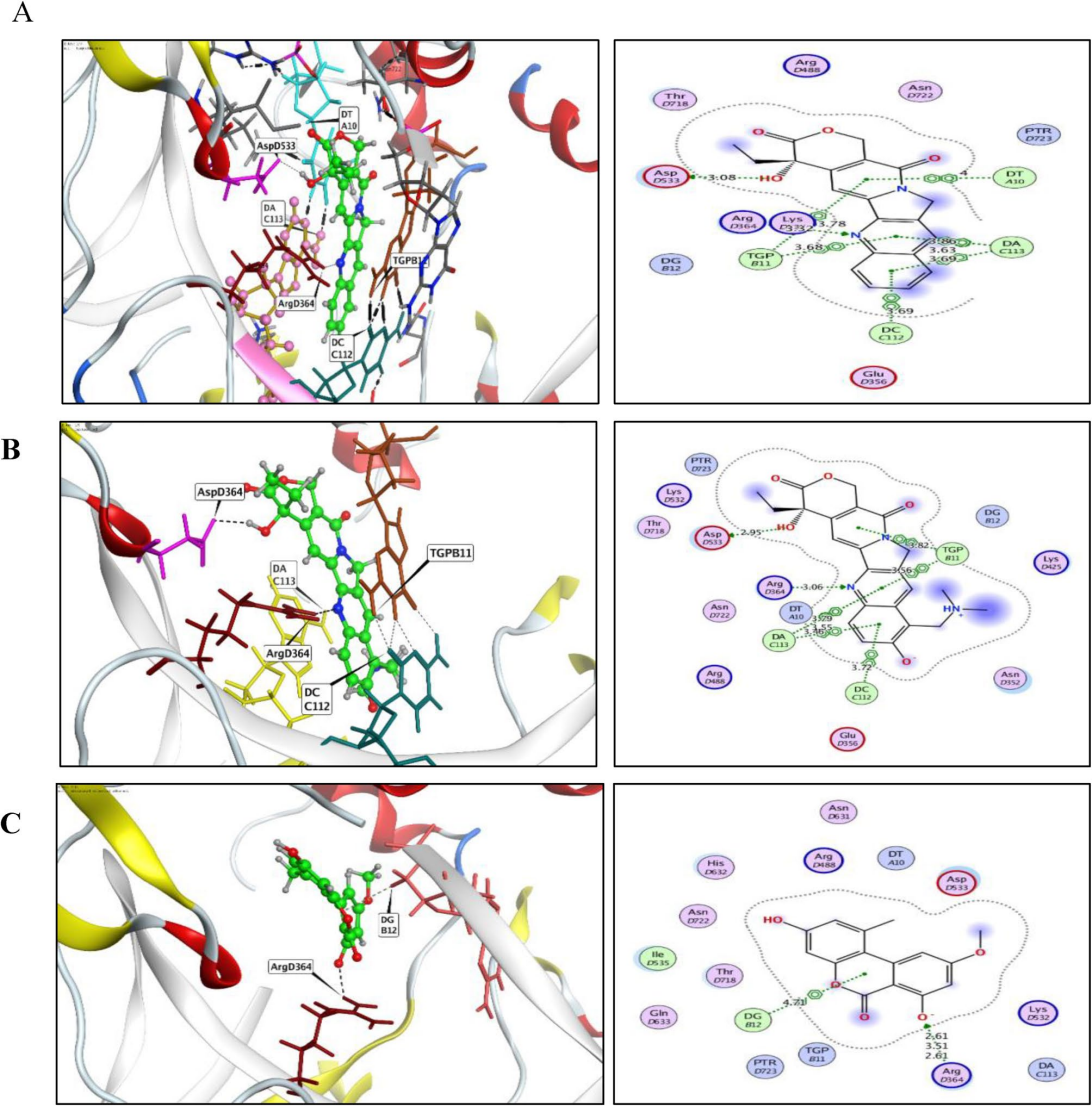
The three -dimensional (right panel) and two-dimensional (left panel) structure of Topoisomerase I docked with Camptothecin (**A**), Topotecan (**B**) and AME (**C**). The green sticks refers to H-bonding, black dashed lines, refers to the topoisomerase I active binding site.

From the docking analysis of AME with topoisomerase I, the binding energy of AME with topoisomerase I active sites was −7.72 kcal/mol, while, the topotecan and camptothecin had the same interaction pattern, with binding energy scores of −9.95 −9.44 kcal/mol (Table 1). The AME has the same molecular interactions of camptothecin and topotecan with one hydrogen bonding with key amino acid Arg364 as a hydrogen bond acceptor and another two ionic bonds with Arg364 and one hydrophobic interaction with DG12 DNA nucleobase (Fig. 5). From the molecular docking analysis of the compounds with topoisomerase II, Etoposide and AME of *A. alternata* have mostly the same binding energies −6.06 and −6.82 kcal/mol with the enzyme active sites domains, respectively. The molecular interactions of Etoposide and AME with topoisomerase II were reported via hydrogen bonding with the two key amino acids Arg503 and Asp479 amino acid residues with RMSD 1.08 Å and 1.96 Å, respectively. The docking results of etoposide, topotecan, and AME of *A. alternata* were summarized in Table 2. Additionally, the AME interaction was exhibited by two hydrogen bonds with the two key amino acids H_2_O-assisted Arg503 and Asp479 amino acid residues as hydrogen bond acceptor moiety, in addition to two H_2_O-assisted hydrogen bonds with DG10 (DNA nucleobase) and Ser480 residue and one hydrophobic interaction with (DT9) DNA nucleobase, with RMSD and binding energy score values of 1.96 Å and −6.06 kcal/mol, respectively (Fig. 5). So, from the *in silico* analysis, the putative compound AME had the same binding pattern of energy and amino acids of Etoposide with the topoisomerase II active site, that unlike to the interaction mode of etoposide with topoisomerase II active sites.

**Table 1:** Molecular modeling of AME, topotecan, and camptothecin at the active binding groove of topoisomerase I protein (PDB code: 1T8I).

Compound	∆G Score Kcal/mol	RMSD_Refine (Å)	Amino Acid/Bond	Distance (Å)
AME	−7.72	1.84	Arg364/H acceptor Arg364/Ionic Arg364/Ionic DG12/pi- H	2.61 3.51 2.61 4.71
Topotecan	−9.95	1.16	Asp533/H donor Arg364/H acceptor TGP11/pi- pi TGP11/pi- pi DA113/pi- pi DA113/pi- pi DA113/pi- pi DC112/pi- pi	2.95 3.06 3.82 3.56 3.79 3.55 3.46 3.72
Camptothecin	−9.44	1.47	Asp533/H donor Arg364/H acceptor DT10/pi- pi TGP11/pi- pi TGP11/pi- pi DA113/pi- pi DA113/pi- pi DA113/pi- pi DC112/pi- pi	3.08 3.32 4.00 3.78 3.68 3.86 3.63 3.69 3.96

**Table 2:** Molecular modeling of AME, topotecan, and Etoposide at the active binding groove of topoisomerase II protein (PDB code: 3QX3).

Compound	∆G Score Kcal/mol	RMSD_Refine (Å)	Amino Acid/Bond	Distance (Å)
AME	−6.06	1.96	Asp479/H acceptor HOH-Arg503/H acceptor HOH-DG10/H acceptor HOH-Ser480/H acceptor DT9/pi- H	3.32 2.83 2.83 2.83 4.32
Topotecan	−7.25	1.54	Arg503/H donor Asp557/H donor HOH-Ser480/H acceptor HOH-DG10/H acceptor MG1222/Metal MG1222/Ionic DT9/pi- H	3.04 3.14 3.33 3.33 2.55 2.55 4.15
Etoposide	−6.82	1.08	Arg503/H donor Asp479/H donor	3.47 3.37

**Fig. 6:**
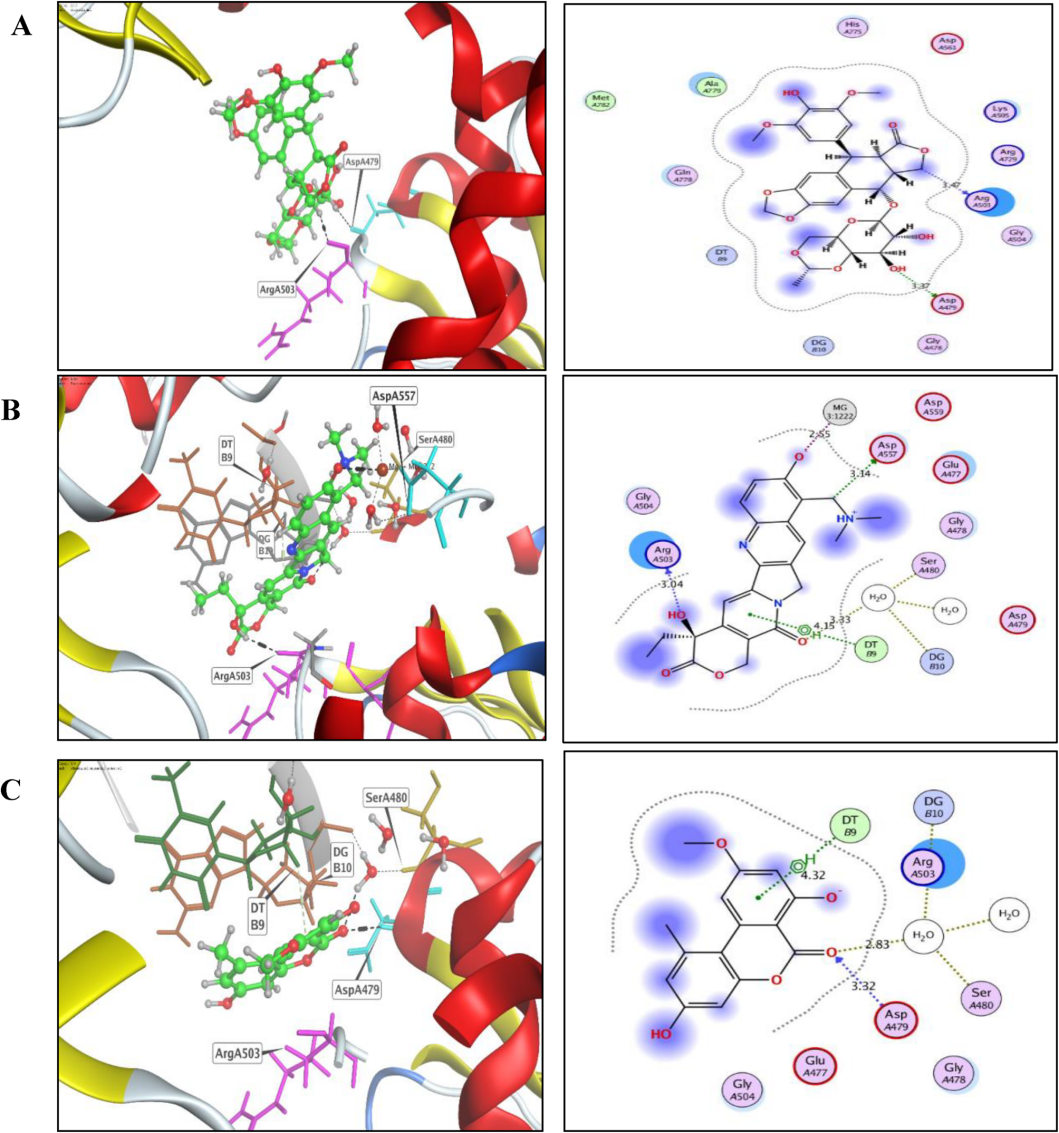
The three -dimensional (right panel) and two-dimensional (left panel) structure of Topoisomerase II docked with Etoposide (**A**), Topotecan (**B**) and AME (**C**). The green sticks refers to H-bonding, black dashed lines, refers to the topoisomerase I active binding site.

From the dynamic simulations, the two clusters show the movement of the NMA, while the two-colored arrows in each model indicate the domain’s mobility direction relied on the results (Fig. 6). The characteristic deformation of each residual side (Cα atom), indicated by chain’s-colored hinges, is the major factor illustrating the complex’s deformability. The eigenvalues were calculated 6.660000^−06^ and 1.0191940^−05^. Furthermore, for each normal mode, the eigenvalue and the variance display a converse relationship (Fig. S2,3). The B-factor plot affords the stable structure coupled with the molecules; the B-factor graph shows the average RMS. The covariance matrix is a color-coded representation, indicating correlated, uncorrelated, and anti-correlated motions in red, white, and blue, respectively. The docked protein molecule’s atoms (Cα) are connected thclass of dibenzopyrone derivatives includes alternariol, AME and altenuenerough “springs” of variable strengths, represented by the darker grey areas in the elastic network model, signifying the stronger springs.

## Discussion

AME is one of the most common dibenzopyrone derivatives of *Alternaria* mycotoxins, with a potential biological activity^27^. However, the productivity and mechanism of action of this compound remains equivocal, thus, chemical and biological characterization of AME in addition to the molecular docking and dynamic simulations analysis were explored. *Alternaria alternata* PV342518.1*,* an endophyte of *C. roseus,* exhibited the highest AME producing potency (400 μg/l). *Alternaria* species were reported to produce a plethora of mycotoxins with a host-specific and non-host-specific activity^45–47^. The non-host-specific toxins produced by *Alternaria* species includes tentoxin, alternaric acid, alternariol, AME, brefeldin A, altertoxins, tenuazonic acid and curvularin^48^ (Meena and Samal, 2019). The secondary metabolites of *Alternaria* were divided into 5 classes a that being coincident with the results of macrophagess reviewed by^49^, the 1^st^ class of dibenzopyrone derivatives includes alternariol, AME and altenuene^50,48^. The identity of *A. alternata* AME was verified by HPLC and LC-MS/MS analyses. The TLC-extracted AME were assessed by HPLC, gave the same retention time of authentic AME, confirming their identity as AME. The chemical structure of the compound was verified by LC-MS analysis, with molecular mass to charge ratio 274.2 *m/z* that was typical to the authentic one. Furthermore, the parent AME molecule (C_15_H_12_O_5_) of *A. alternata* had the same fragmentation pattern of the authentic AME, with fragments of 256.2, 105.9, 87.9, 70.0, 69.9, 56.9, 42.9 and 41.7 m/z, ensuring their chemical identity as AME^51^.

The purified *A. alternata* AME had a significant antiproliferative activity against the cells MCF-7, HepG-2 and Caco2, compared to OEC cells, in a concentration-dependent pattern. The selectivity index of the purified *A. alternata* AME was 3.6–3.9.6.9 folds for the tested tumor cells, compared to the normal OEC ones. The IC_50_ values of purified *A. alternata* AME against Topoisomerase I and II were 16.7 nM and 10.2 nM, respectively. So, the affinity of *A. alternata* AME to bind with topoisomerase II was 1.64 folds more than Topoisomerase I, as consistent with the previous studies^27,52^. Practically, the AME received less attention regarding to its activity as topoisomerases inhibitor, compared to alternariol. Alternariol has been recognized by its affinity to inhibits DNA topoisomerase I and II, with a selectivity for IIa isoform^27,52,52^, stabilizing the covalent topoisomerase DNA intermediates (topoisomerase poison). From the cell cycle analysis of MCF-7 cells in response to *A. alternata* AME the highest growth arrest was observed at G2/M phase by 96.4% reduction in growth compared to 82.5 % reduction of control cells, i.e 1.2 folds. The putative compound had an obvious inhibitory effect on the cell cycle of MCF-7 at their S-phase by 2 folds, compared to the control cells. From the cell cycle, AME of *A. alternata* had a significant inhibitory effect to the cells at their G2/M and S phases, confirming the interference with the cellular of machinery of mitotic division and DNA synthesis, that being coincident with the results of macrophages^53–55^. Consistently, alternariol reduces the cell cycle at the S phase with a noticeable arrest to the cells at the G0/G1 phase^56^ of the porcine endometrial cancer cells. The reduced proliferation is in most cases accompanied by accumulation of cells in the G2/M-phase^53–55^. As well as, alternariol could activate p53, that play a central role in regulation of DNA repair, cell cycle arrest, apoptosis, and autophagy^57^. From the flow cytometry, the AME of *A. alternata* had a significant effect on inducing the total apoptosis, early apoptosis and necrosis of MCF-7 by 20, 22 and 2.9 folds, compared to control cells. Strikingly, with AME, the late apoptosis of MCF-7 cells was increased by 228.6 folds, compared to the control cells.

The molecular affinity of the AME of *A. alternata* to bind with Topoisomerase I and II was assessed from the docking analysis. From the in docking analysis with topoisomerase I active site, the topotecan and camptothecin had the same interaction pattern with binding energy scores of −9.95 and −9.44 kcal/mol, while the binding energy of AME was −7.72 kcal/mol, with the same molecular interaction at the key amino acid Arg^364^. From the docking results with topoisomerase II active sites, Etoposide and AME have the same binding energies (−6.06 and −6.82 kcal/mol), with the molecular interactions via the hydrogen bonding of Arg^503^ and Asp^479^ amino acid residues. So, from the *in silico* analysis, the AME had the same binding energies, amino acids pattern of Etoposide with topoisomerase II active site. The molecular dynamic simulations of the AME with topoisomerase I and II was conducted to evaluate the structural dynamics and molecular mobility of these complexes. From the dynamic simulations, the two clusters show the movement of the normal Mode Analysis (NMA), while the two-colored arrows in each model indicate the domain mobility direction. The covariance matrix is a color-coded representation, indicating correlated, uncorrelated, and anti-correlated motions in red, white, and blue, respectively.

In conclusion, *A. alternata,* an endophyte of *Strelitzia nicolai,* morphologically identified and molecularly confirmed relied on their ITS sequence with accession # PV342518.1, was reported as a potential AME producer. The chemical structure of the putative AME was resolved from the HPLC and LC-MS/MS analyses. The cytotoxic activity of the recovered AME was assessed towards the different cell lines, compared to the OEC as normal cells. The purified *A. alternata* AME had a significant activity to inhibit the Topoisomerase II than topoisomerase I, with a potential activity to triggers the apoptosis process, and halting the cell cycle at the G2/M phase and S-phase. The docking analysis of AME with the topoisomerases I (1T8I) and II demonstrate their ability to adopt the main bio-molecular interactions relative to those of camptothecin and etoposide, respectively. The molecular dynamics study emphasized that AME can make stable interactions with topoisomerases I and II proteins, additional establishment of this stability indicated by B-factor and deformability models. So, with further experimental, metabolomic and computational analyses, AME of *A. alternata* could be a novel candidate with higher anticancer activity via mainly inhibiting the mitotic machinery and DNA synthesis of the tumor cells.

## Supplementary Information

Supplementary Information.

## Data Availability

All the data are provided in the manuscript.
