# Supplementary material for: Characterization of Alternaria alternata alternariol monomethyl ether with a potential antiproliferative activity by topoisomerases inhibition; molecular docking and dynamic simulations
Source: Sci Rep. 2026 May 18;16:15352. doi: 10.1038/s41598-026-51757-8 (PMC13184112; doi:10.1038/s41598-026-51757-8)
Supplement: Supplementary file 1 — Supplementary Information. [file 41598_2026_51757_MOESM1_ESM.pdf]

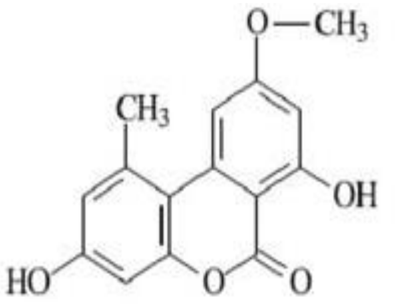

Alternariol monomethyl ether

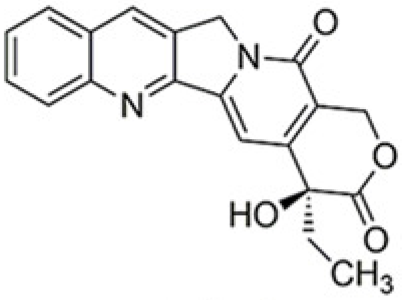

Camptothecin

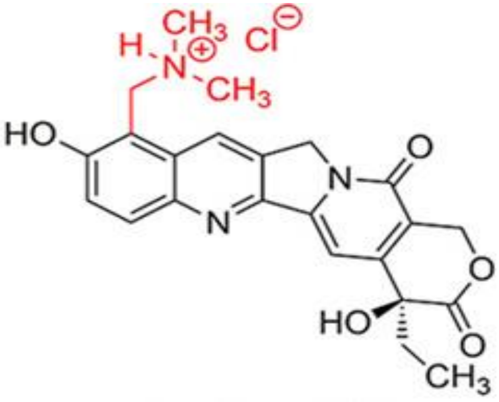

Topotecan

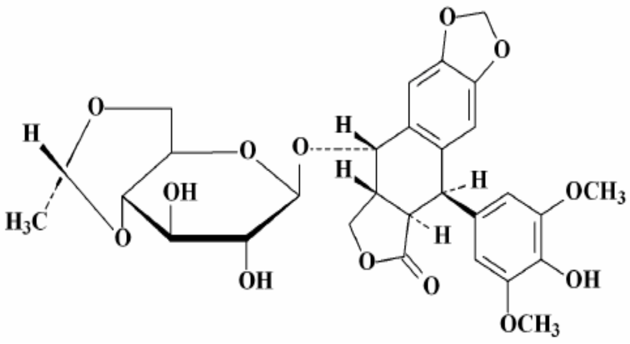

Etoposide

Fig. S1. The chemical structures of Alternariol monomethyl ether, Camptothecin, Topotecan and Etoposide

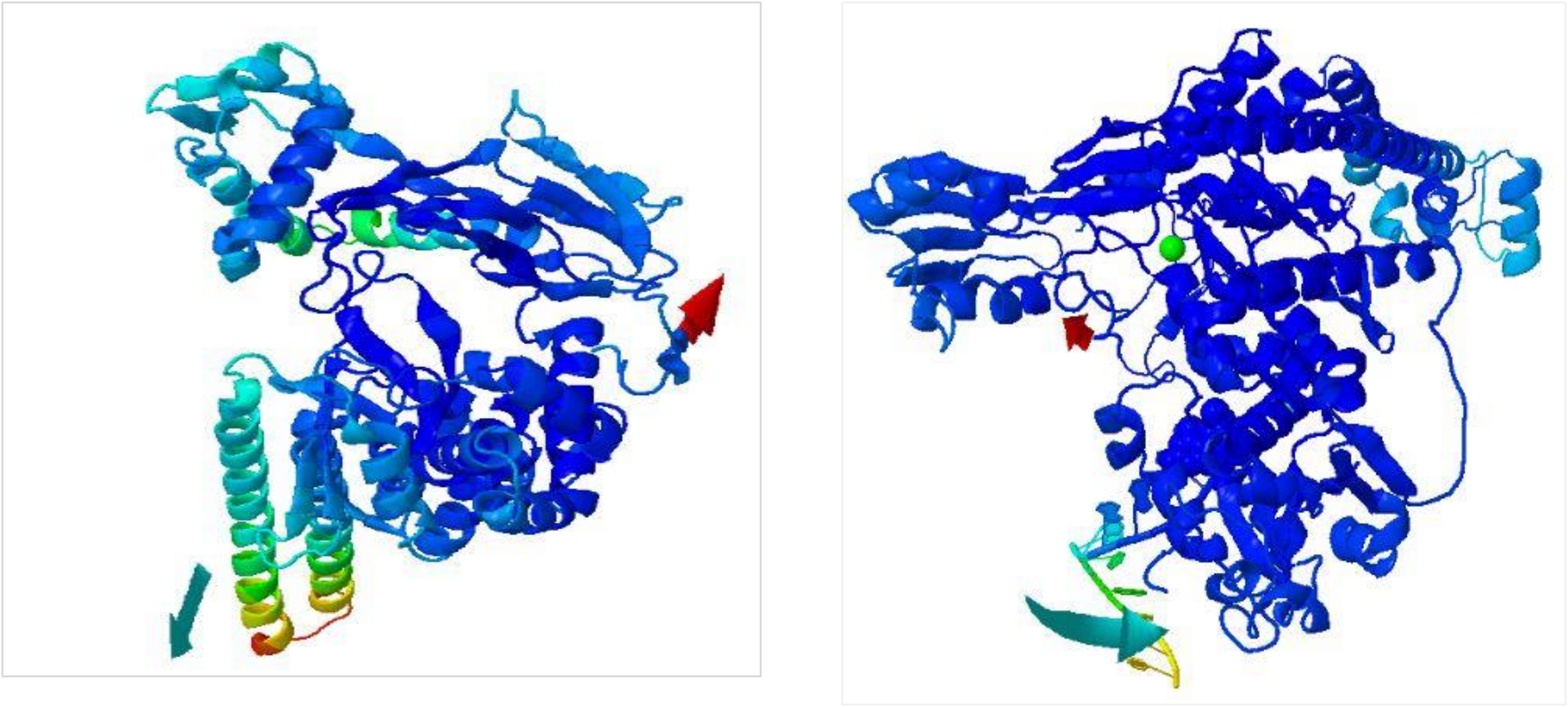

Fig. S2. The docked complexes (1T8I- alternariol monomethyl ether) and (3QX3- alternariol monomethyl ether) normal Mode Analysis (NMA) models assessing the molecular mobility. (Two-colored affine arrows show the domain's mobility direction).

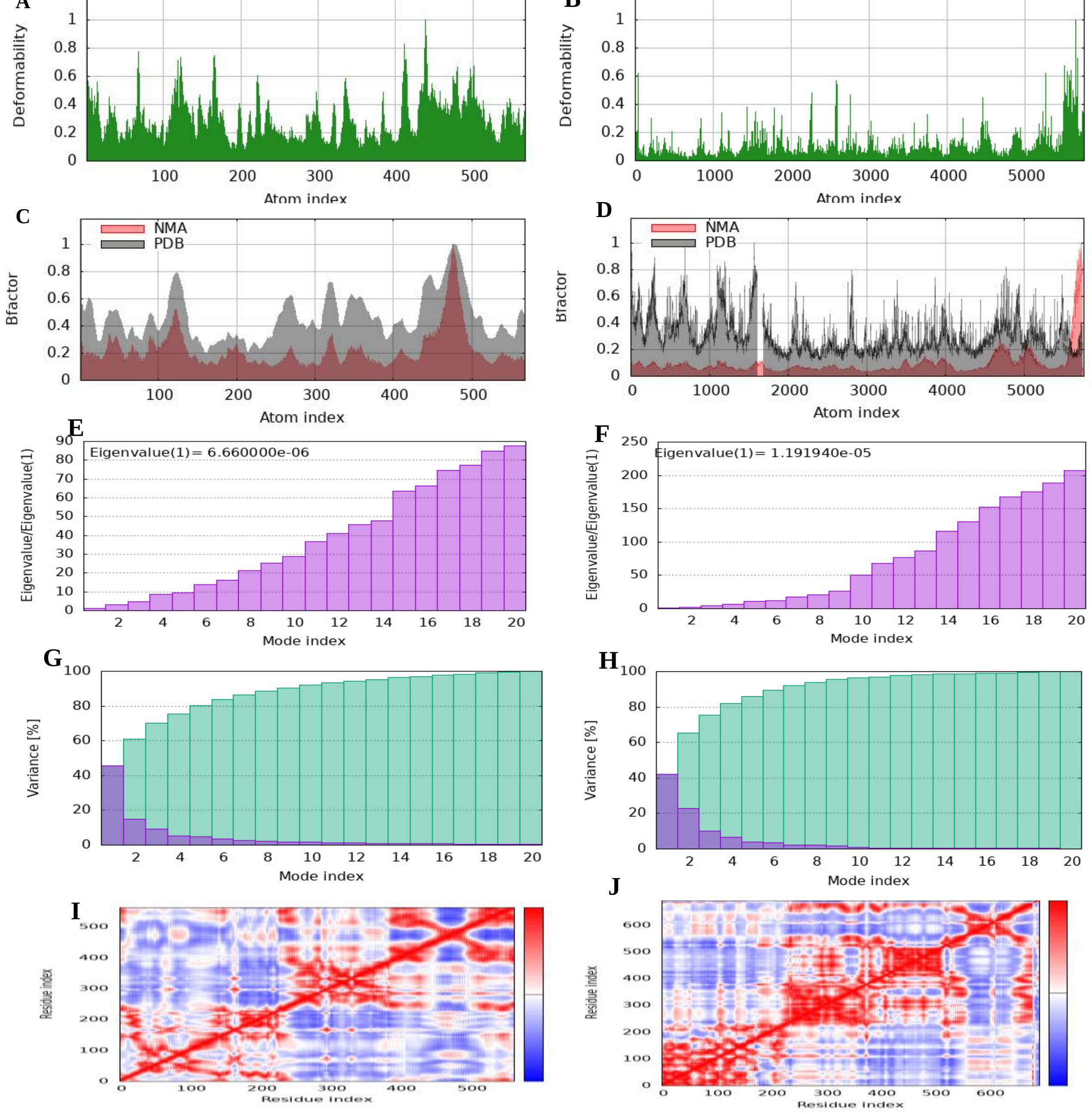

Fig. S3. The analyzed outcomes (via iMODS website) of the docked complexes (1T8I, AME) and (3QX3- AME), as the following: A&B deformabilitycharts, C&D B-factor plots, E&F eigenvalues, G&H variance plots, I&J covariance matrix analysis, and K&L elastic systems.
